# Preclinical study and parallel phase II trial evaluating antisense STAT3 oligonucleotide and checkpoint blockade for advanced pancreatic, non-small cell lung cancer and mismatch repair-deficient colorectal cancer

**DOI:** 10.1136/bmjonc-2023-000133

**Published:** 2024-07-30

**Authors:** Chad Tang, Genevieve P Hartley, Coline Couillault, Ying Yuan, Heather Lin, Courtney Nicholas, Anupallavi Srinivasamani, James Dai, Ecaterina E Ileana Dumbrava, Siqing Fu, Daniel D Karp, Aung Naing, Sarina A Piha-Paul, Jordi Rodon Ahnert, Shubham Pant, Vivek Subbiah, Timonthy A Yap, Apostolia M Tsimberidou, Paola Guerrero, Sarah Dhebat, Theresa Proia, Michael A Curran, David S Hong

**Affiliations:** 1 GU Radiation Oncology, The University of Texas MD Anderson Cancer Center, Houston, Texas, USA; 2 Department of Investigational Cancer Therapeutics, University of Texas MD Anderson Cancer Center, Houston, Texas, USA; 3 Department of Translational Molecular Pathology, The University of Texas MD Anderson Cancer Center, Houston, Texas, USA; 4 Department of Immunology, The University of Texas MD Anderson Cancer Center, Houston, Texas, USA; 5 Department of Biostatistics, The University of Texas MD Anderson Cancer Center, Houston, Texas, USA; 6 University of Texas Health Science Center at Houston Graduate School of Biomedical Science, Houston, Texas, USA; 7 Oncology R&D, Research & Early Development, AstraZeneca PLC, Waltham, Massachusetts, USA

**Keywords:** Pancreatic cancer, Immunotherapy, Lung cancer (non-small cell)

## Abstract

**Objective:**

To evaluate signal transducer and activator of transcription 3 (STAT3) inhibition we conducted a co-clinical trial testing danvatirsen, a STAT3 antisense oligonucleotide (ASO) and checkpoint inhibition in conjunction with preclinical experiments.

**Methods and analysis:**

Orthotopically implanted pancreatic cancer (pancreatic adenocarcinoma (PDAC)) was treated with STAT3 ASO with immune checkpoint inhibition. Tumour infiltrating immune cell populations were characterised via flow cytometry. In vitro experiments evaluated STAT3 inhibition in pancreatic stellate cells (PSCs) and myeloid-derived suppressor cells (MDSCs).

A phase II trial employing a Simon II stage design tested the clinical efficacy of danvatirsen and durvalumab in non-small cell lung cancer (NSCLC), PDAC and mismatch repair-deficient colorectal cancer (MRD CRC). The primary objective was 4-month disease control rate (DCR).

**Results:**

In vivo studies identified improvement in survival of PDAC implanted mice treated with STAT3 ASO and checkpoint inhibition. Within tumour-infiltrating lymphocytes there was expansion of CD4 and PD-1+ CD8 populations with STAT3 ASO.

Thirty-seven patients (29 PDAC, 7 NSCLC and 1 MRD CRC) from a single institution started treatment on trial between April 2017 and March 2020. No objective responses were observed. Four of six (66.7%, 95% CI 22.3% to 95.7%) NSCLC and 4 of 23 (17.4%, 95% CI 5% to 38.8%) PDAC patients exhibited 4-month DCR. Follow-up in vitro studies revealed an anti-inflammatory and pro-tumour effect of STAT3 ASO mediated by PSCs and MDSCs distinct from ablation of STAT3.

**Conclusion:**

Although durvalumab and danvatirsen met the primary endpoint, no objective responses were observed. A rationale for the lack of objective responses is danvatirsen-induced myeloid immune suppression.

**Trial registration number:**

NCT02983578.

**WHAT IS ALREADY KNOWN ON THIS TOPIC**

- Signal transducer and activator of transcription 3 (STAT3) is a promoter of neoplastic progression through multiple mechanisms including immune suppression.
- Danvatirsen is an antisense oligonucleotide (ASO) that inhibits STAT3. Danvatirsen monotherapy has shown antitumour activity in multiple tumour types.
- In preclinical models danvatirsen has demonstrated synergy with anti-PD-L1 inhibition.

**WHAT THIS STUDY ADDS**

- In phase II clinical trial, combination danvatirsen and durvalumab (anti-PD-L1) led to the study primary endpoint of 4-month disease control in four of six (66.7%, 95% CI 22.3% to 95.7%) patients with non-small cell lung cancer (NSCLC) and 4 of 23 (17.4%, 95% CI 5% to 38.8%) patients with pancreatic cancer. No objective responses were observed.
- Preclinical evidence suggests that STAT3 ASO may enhance suppressive myeloid and fibroblast function rather than purely inhibit STAT3 function.

**HOW THIS STUDY MIGHT AFFECT RESEARCH, PRACTICE OR POLICY**

- Despite not being fully accrued, the lower bounds of the 95% CI exceed the null 4-month disease control rate for both NSCLC and pancreatic adenocarcinoma cohorts, suggesting that this combination should be studied further.
- Future trials employing STAT3 inhibition may achieve greater efficacy if rational combinations are developed or the drug is modified to reduce myeloid-derived immunosuppressive effects.

## Introduction

 Since the emergence of immune checkpoint inhibitors for cancer treatment, there has been interest in therapeutic targets outside of PD-1/L1 and CTLA-4. One candidate is STAT3 (signal transducer and activator of transcription 3), a ubiquitous transcription factor and multifaceted regulator of immune suppression expressed in cancers and surrounding stroma. Constitutive STAT3 expression induces neoplastic transformation and tumorigenesis, driving cancer development, maintenance of stemness and promotion of tumorigenicity.^1^,^5^ A major component of STAT3 activity is its multifaceted promotion of immune suppression, which includes activation of myeloid-derived suppressor cell (MDSC) function and tumour-supportive macrophage polarisation. STAT3 has emerged as a promising drug target, and anti-STAT3 agents have demonstrated promising therapeutic activity across cancer models including non-small cell lung cancer (NSCLC), mismatch repair-deficient colorectal cancer (MRD CRC) and pancreatic adenocarcinoma (PDAC).^6^,^9^ Parallel to STAT3-mediated immune suppression exist other pathways including PD-1/PD-L1, a clinically relevant pathway that produces multiple inhibitor signals including T cell inhibition. Multiple drugs have been developed to target this pathway including the PD-L1 inhibitor durvalumab.^10^

Danvatirsen (AZD9150) is a 16-nucleotide antisense oligonucleotide (ASO) that downregulates STAT3 expression.^6^ Danvatirsen monotherapy has demonstrated clinical activity in multiple cancer types, including NSCLC, lymphoma, and head and neck squamous cell carcinoma.^6 11^ Evaluation of tumour biopsies from clinical trials (NCT01563302 and NCT01839604) revealed selective uptake of danvatirsen into the tumour microenvironment with the potential to reverse immune suppression.^12^ In preclinical models danvatirsen enhanced the efficacy of PD-L1 blockade, demonstrating in vivo synergy.^12^ We conducted a co-clinical trial and basic science experiment to test the efficacy of combination STAT3 inhibition and checkpoint blockade. In the laboratory component, we evaluated the therapeutic potential of a murine STAT3 ASO to potentiate the effects of combination with checkpoint blockade in an orthotopic model of PDAC. In parallel, we conducted a phase II clinical trial evaluating the combination of danvatirsen and checkpoint blockade in cancers that have previously demonstrated efficacy with STAT3 blockade: PDAC, NSCLC and CRC.^6^,^9^

## Materials and methods

### In vivo survival studies

The aggressive metastatic mT4 model of PDAC was developed from *Pdx1-Cre; Kras+/LSL-G12D; Trp53+/LSL-R172H* (KPC) mouse organoids by the Tuveson Laboratory.^13^ The derivative luciferase-expressing mT4-Lyt2-Luc line was created through retroviral transduction of mT4-2D with firefly luciferase linked to a truncated CD8 reporter (Lyt2). Tumour cells were clonally selected and expanded to form the aggressive mT4 Lyt2-luc LA cell line.

Male C57BL/6J, B6-albino, and LysM^Cre/Cre^ Stat3^flox/flox^ mice (Jackson Laboratories) were orthotopically implanted with 3.5×10^4^ mT4 Lyt2-luc LA cells in 30% collagen matrix (Matrigel, BD) in the pancreas on day 0, and LysM^Cre/Cre^ Stat3^flox/flox^ mice were implanted similarly with 3.5×10^4^ mT4 Lyt2-luc LA cells. Mice were treated with anti-CLTA-4 (clone 9H10, 100 µg/100 µL intraperitoneally; Bio X Cell) and/or anti-PD-1 (clone RMP1-14, 250 µg/100 µL intraperitoneally; Leinco) antibodies. Murine STAT3 ASO (AstraZeneca) and control ASO (Ionis Pharmaceuticals) were administered at 50 mg/kg subcutaneously for in vivo treatments and used at 5 µM and 20 µM for in vitro studies.

For in vivo flow cytometric analyses of tumours, mice were implanted similarly with 1×10^5^ mT4 Lyt2-luc LA cells and treated on days 10, 13 and 16 (100 µL intraperitoneally) with the indicated antibodies (online supplemental table S1) and/or with ASO on days 10–17 (100 µL subcutaneously). For flow cytometry analyses, mice were euthanised for tumour harvest on day 18, and tissues were processed to isolate tumour-infiltrating lymphocyte (TIL) populations. For in vivo survival studies mice were euthanised on becoming moribund or when tumour volume exceeded 1000 mm^3^.

### Clinical trial drug administration

Patients underwent a 7-day lead-in phase, during which danvatirsen was administered on days 1, 3 and 5. Following the lead-in phase, treatment was administered in 4-week cycles in which durvalumab was given on day 1 of each cycle and danvatirsen was administered weekly. Danvatirsen and durvalumab were administered intravenously at 3 mg/kg ideal body weight and 1500 mg fixed dose, respectively. Dose reductions were not allowed. Treatments were continued until the occurrence of progressive disease, death, adverse events requiring treatment cessation or withdrawal of consent.

### Clinical trial design

This trial was a single centre, open-label, phase II study consisting of three independent patient cohorts: NSCLC, PDAC and MRD CRC. The trial employed a two-stage design modified from a Simon 2-stage design.^14^ The primary outcome was 4-month disease control rate (DCR). Disease control (DC) was defined as achievement of partial response, complete response or stable disease (SD) lasting at least 4 months from initiation of the first lead-in cycle.

### Adverse events on clinical trial

Adverse events were collected starting from full enrolment to study withdrawal/completion or end of follow-up period. Adverse event severity was graded using CTCAE version 4.03 by the treating physician. An adverse event was considered ‘related’ to study drug treatment if the association was judged to be possibly, probably or definitely related.

### Translational correlative studies

Samples for translational correlative studies were collected if deemed safe and clinically feasible. In lieu of available archived formalin-fixed, paraffin-embedded tissues, a baseline pretreatment biopsy sample was obtained. Pretreatment biopsy samples were assessed for mutations using a CLIA-certified gene panel containing 70–200 genes, which was developed for solid tumours at our institution.^15^

Peripheral blood for translational correlatives was obtained during screening, at the end of the first cycle of treatment, and up to 1 month after the last cycle of systemic therapy. *KRAS* mutation status from the peripheral blood was assessed in 12 pancreatic patients via cell free DNA (cfDNA) and exosomal DNA. Plasma was isolated as previously described.^16^ Droplet digital PCR (ddPCR) (QX200, Biorad) was used for detection of KRAS mutants as previously described.^16^

### Statistics

A two-stage design was used for the NSCLC and pancreatic cancer cohorts independently. During the first stage, 12 patients would have been enrolled by design for each cohort. Accrual suspension should occur until ≥3 patients and ≥1 patient for NSCLC and pancreatic cancer, respectively demonstrate DC. During the second stage, an additional 18 patients would be enrolled for each cohort. In the mismatch repair-deficient CRC cohort, 15 patients were planned to be enrolled without interruption. This trial was reviewed by the MD Anderson Cancer Center Institutional Review Board, and consent was obtained for all patients.

For the NSCLC cohort, the target undesirable and desirable 4-month DCR was P_0_≤20% and P_1_≥40%, respectively. The combination would be declared worthy of further study in the NSCLC cohort if at least 9 of 30 patients demonstrated DC at 4 months. The two-stage design yields 86% power with one-sided alpha of 11%. There is a 56% chance of stopping early if the true 4-month DCR was 20%. For the pancreatic cancer cohort, the target undesirable and desirable 4-month DCR was P_0_≤5% and P_1_≥20%, respectively. The combination would be declared worthy of further study in the pancreatic cancer cohort if at least 4 of 30 patients exhibited 4-month DC. The two-stage design yields 84% power with one-sided alpha of 5.5%. There is a 54% chance of stopping early if the true success rate was 5%. Given the low anticipated accrual in the MRD CRC cohort, no formal success threshold and power calculation was conducted a priori.

For laboratory and translational analyses, statistical comparisons between data sets with two treatment groups were done using the Mann-Whitney U test. Comparisons between three or more groups were done using analysis of variance (ANOVA), followed by Tukey multiple means post-test. Analyses were done using Prism software (GraphPad). Statistical comparisons for in vivo survival and clinical trial analyses were done with Kaplan-Meier and log-rank (Mantel-Cox) tests or multiple t-tests with Bonferroni correction.

### PPI statement

Given the advanced nature of the cancer in patients enrolled on the trial, DC represents a meaningful endpoint. Patients were not involved in this design nor advertising for recruitment. We have and will continue to discuss clinic trial results and their implications with study participants.

## Results

### Preclinical outcomes

#### Multimodal therapy of STAT3 ASO combined with anti-CTLA-4 and anti-PD-1 antibodies prolongs survival in aggressive mT4-LA PDAC model

**Figure 1:**
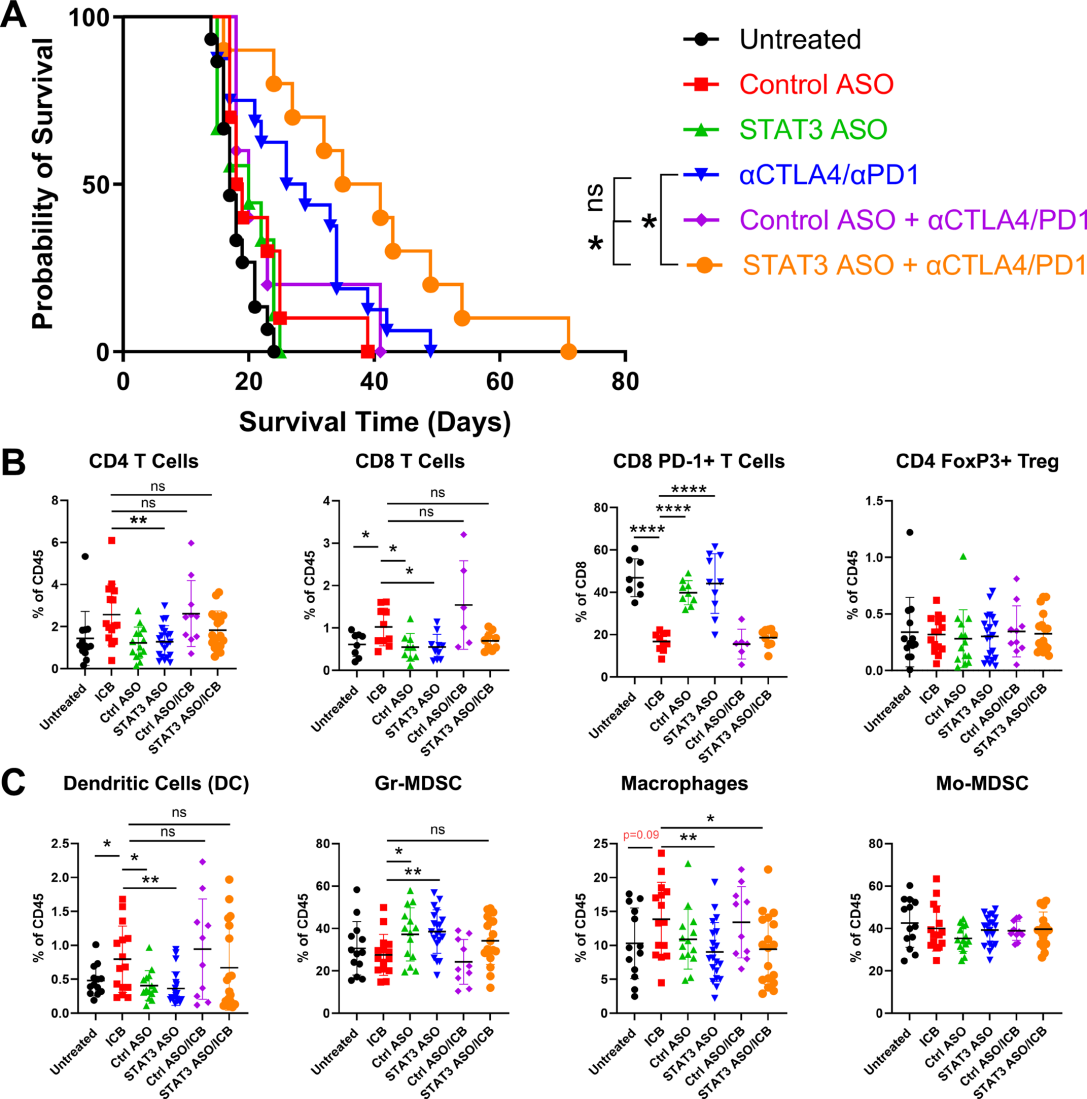
STAT3 ASO and anti-CTLA-4 and anti-PD-1 antibodies combination therapy prolongs survival in the mT4 pancreatic tumour model, but STAT3 ASO alone favours a pro-tumorigenic environment. (**A**) 5- to 8-week-old mice were implanted with 3.5×10^4^ mT4 Lyt2-luc LA cells orthotopically and were treated on days 10, 14, 18, 22, 26 and 30 (intraperitoneally) with the indicated antibodies and/or with ASO on days 10–30 (subcutaneously). For in vivo survival experiments, mice were euthanised if moribund or when tumour volume exceeded 1000 mm^3^. Kaplan-Meier curves show overall survival. Data are cumulative of n=4 experiments, with 10–15 mice per group. Mice were implanted with 1×10^5^ mT4 Lyt2-luc LA cells and treated on days 10, 13 and 16 (100 µL intraperitoneally) with the indicated antibodies and/or with ASO on days 10–17 (100 µL subcutaneously). For flow cytometry experiments, mice were euthanised for tumour harvest on day 18, and tissues were processed for (**B**) tumour-infiltrating lymphocyte or (**C**) granulocyte populations. Data shown are cumulative of n=2 experiments, with 10–15 mice per group. Statistical significance for (**A**) was calculated using the Gehan-Breslow-Wilcoxon test, with *p<0.05. For (**B and C**), statistical significance was calculated using one-way analysis of variance followed by Tukey multiple means post-test, with *p<0.05, **p<0.005 and ****p<0.0001. ASO, antisense oligonucleotide; DC, dendritic cell; Gr, granulocytic; ICB, immune checkpoint blockade; MDSC, myeloid-derived suppressor cell; Mo, monocytic; STAT3, signal transducer and activator of transcription 3.

To investigate the impact of STAT3 inhibition and immune checkpoint blockade (ICB) on the suppressive elements of the PDAC tumour microenvironment, mT4 tumours were orthotopically implanted into syngeneic C57BL/6 mice and treated with ICB with anti-CTLA-4 and anti-PD-1 antibodies combined with STAT3 or control ASO. We found that addition of ICB modestly extended survival compared with untreated mice, which was further extended with the addition of the STAT3 ASO (p=0.034) compared with the combination of a control STAT3 ASO, anti-CTLA-4 and anti-PD-1 antibodies or compared with anti-CTLA-4/anti-PD-1 alone (p=0.045) (figure 1A and online supplemental table S2).^12 17^ No survival benefit was seen in mice treated with STAT3 ASO compared with untreated controls.

Combination checkpoint blockade expanded the CD4^+^ T cell compartment compared with control ASO and STAT3 ASO monotherapy (figure 1B). ICB combinations with control or STAT3 ASO were not statistically different from ICB alone. CD8 T cells showed a similar pattern of upregulation in response to ICB combination which did not significantly differ with the addition of either ASO. ICB treatment also resulted in significant downregulation of PD-1 expression, a sign of reduced exhaustion, in CD8 T cells compared with control and STAT3 ASO. Notably, there was an increase in PD-1 expression on CD8 T cells treated with STAT3 ASO, suggesting T cell exhaustion; however, STAT3 ASO and ICB combination therapy showed equivalently low PD-1 levels to ICB alone (figure 1B).

In the myeloid compartment, treatment with anti-CTLA-4 and anti-PD-1 antibodies lead to expansion of dendritic cell populations and contraction of the granulocytic MDSC (GrMDSC) population relative to either control or STAT3 ASO (figure 1C). ICB combinations with control or STAT3 ASO were not statistically different from ICB alone in either setting. ICB combination expanded tumour associated macrophages relative to ASO monotherapy and trending (p=0.09) relative to untreated. This expansion was abrogated when CTLA-4 and PD-1 blockade was combined with STAT3 ASO revealing a potential advantage of the triplet therapy (figure 1C).

#### STAT3 ASO augments immune suppressive pathway expression in MDSCs in vitro

**Figure 2:**
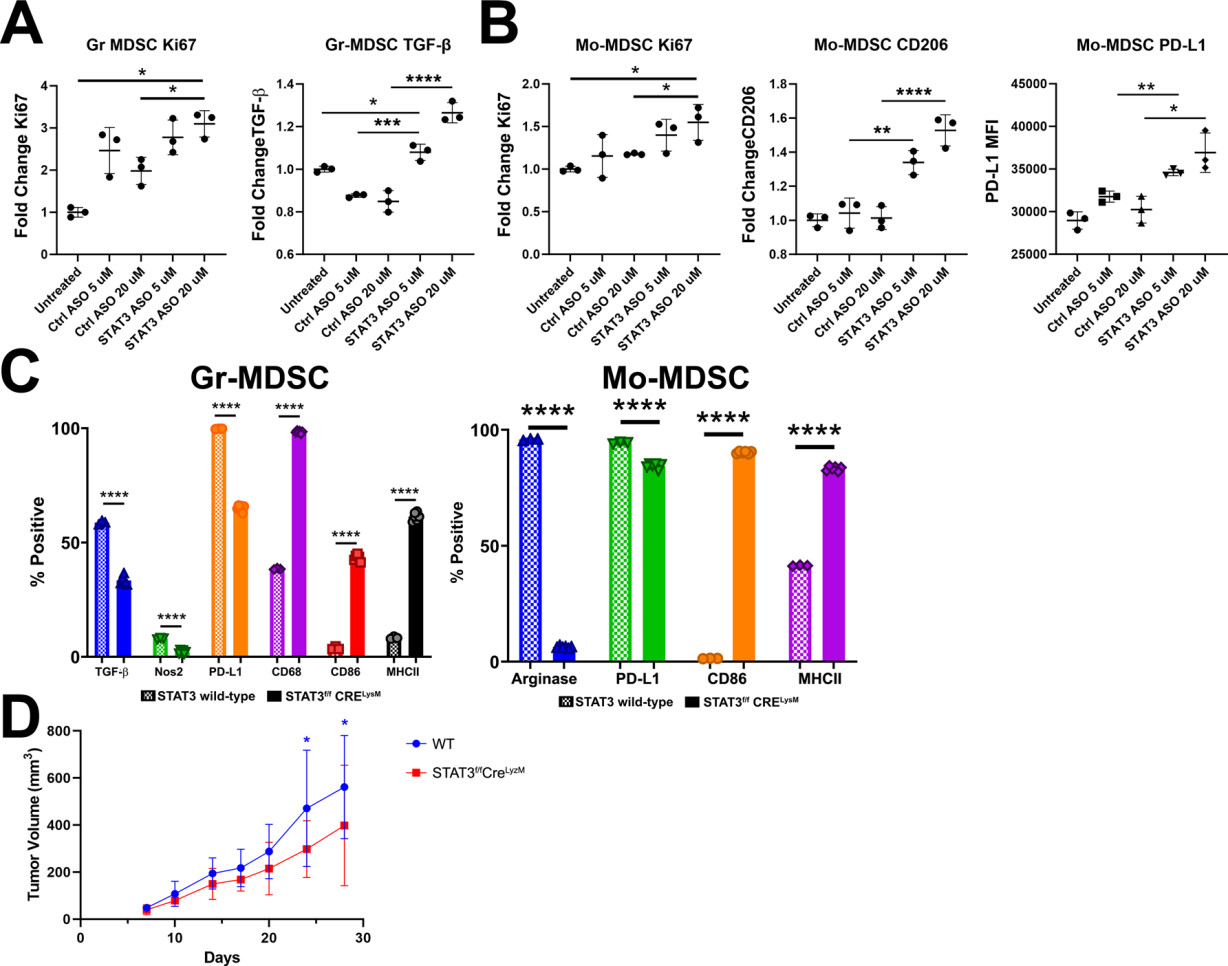
Immune-suppressive effects of STAT3 ASO on MDSC differ from genetic ablation of STAT3. Bone marrow MDSCs were cultured in GM-CSF and IL-6 as well as STAT3 or control ASO for 4 days and then in ASO alone for an additional 4 days and stained for (**A**) TGF-β production by Gr MDSC and (**B**) immune suppressive molecule expression and by monocytic MDSCs. (**C**) MDSCs from wild-type mice (solid) and STAT3^f/f^ Cre^LysM^ mice (hashed bars) were cultured with only GM-CSF and IL-6 and phenotyped similarly for comparison. (**D**) mT4-LA tumour growth in wild-type and STAT3^f/f^ Cre^LysM^ mice was measured without treatment. Data shown are representative of two independent experiments. Statistical significance for (**A**) and (**B**) were calculated using one-way analysis of variance (ANOVA) followed by Tukey multiple means post-test, with *p<0.05, **p<0.005, ***p<0.0005 and ****p<0.0001. Statistical significance for (**C**) were calculated using two-way ANOVA followed by Sidak multiple comparisons post-test, with ****p<0.0001. Statistical significance for survival was calculated using multiple t-tests with Bonferroni correction, with *p<0.05. ASO, antisense oligonucleotide; Gr, granulocytic; IL, interleukin; MDSC, myeloid-derived suppressor cell; MFI, mean fluorescence intensity; Mo, monocytic; STAT3, signal transducer and activator of transcription 3; WT, wild type.

To determine the effect of a STAT3 ASO on MDSC programming, we matured murine bone marrow-derived myeloid cells in the presence of STAT3 ASO and assessed their phenotype by flow cytometry. We found increased GrMDSC proliferation and production of TGF-β (figure 2A). In the monocytic MDSC (MoMDSC) compartment also saw increased proliferation and upregulation of both CD206 and PD-L1 (figure 2B). To validate these findings, we compared untreated bone marrow-derived myeloid cells taken from STAT3^f/f^ Cre^LysM^ mice to wild-type myeloid cells treated with STAT3 ASO. As expected, MDSCs cultured from STAT3^f/f^ Cre^LysM^ mice showed a more pro-inflammatory and less immune suppressive phenotype, in contrast to our observations with the STAT3 ASO (figure 2C). We also compared mT4-Lyt2-Luc-LA tumour burden in wild-type and STAT3^f/f^ Cre^LysM^ mice and found delayed tumour growth in mice that lacked STAT3 expression in myeloid cells (figure 2D).

#### Male and female myeloid cells differ in their response to STAT3 ASO

Recent publications that suggest that MDSC populations differ between male and female mice and that this dimorphism has significant implications on disease progression. MDSCs from male and female mice exhibited dimorphic responses to treatment with STAT3 ASO. There was a trend towards increased numbers of GrMDSCs in male mice whereas this population decreased in female mice, and there was also a significant decrease in the MoMDSC compartment of female mice (online supplemental figure S1A). STAT3 ASO stimulated proliferation of GrMDSCs in male mice at the high concentration of 20 µM, and a similar trend was observed in MoMDSCs, with no changes in MDSC proliferation in female mice (online supplemental figure S1B). Analysis of the MDSC phenotype revealed increased LAP, PD-L1 and CD206 expression in male MoMDSCs (online supplemental figure S1C). In contrast, LAP expression decreased in female MoMDSCs. We also phenotyped macrophages and found that treatment with STAT3 ASO decreased the percentage of macrophages generated from male bone marrow but increased the number from female bone marrow (online supplemental figure S2A). In male mice, these macrophages produced in the context of STAT3 ASO showed expression of LAP and arginase I following treatment with 20 µM STAT3 ASO but showed decreased LAP expression and no change in arginase I in female mice (online supplemental figure S2B).

### Clinical trial outcomes

**Figure 3:**
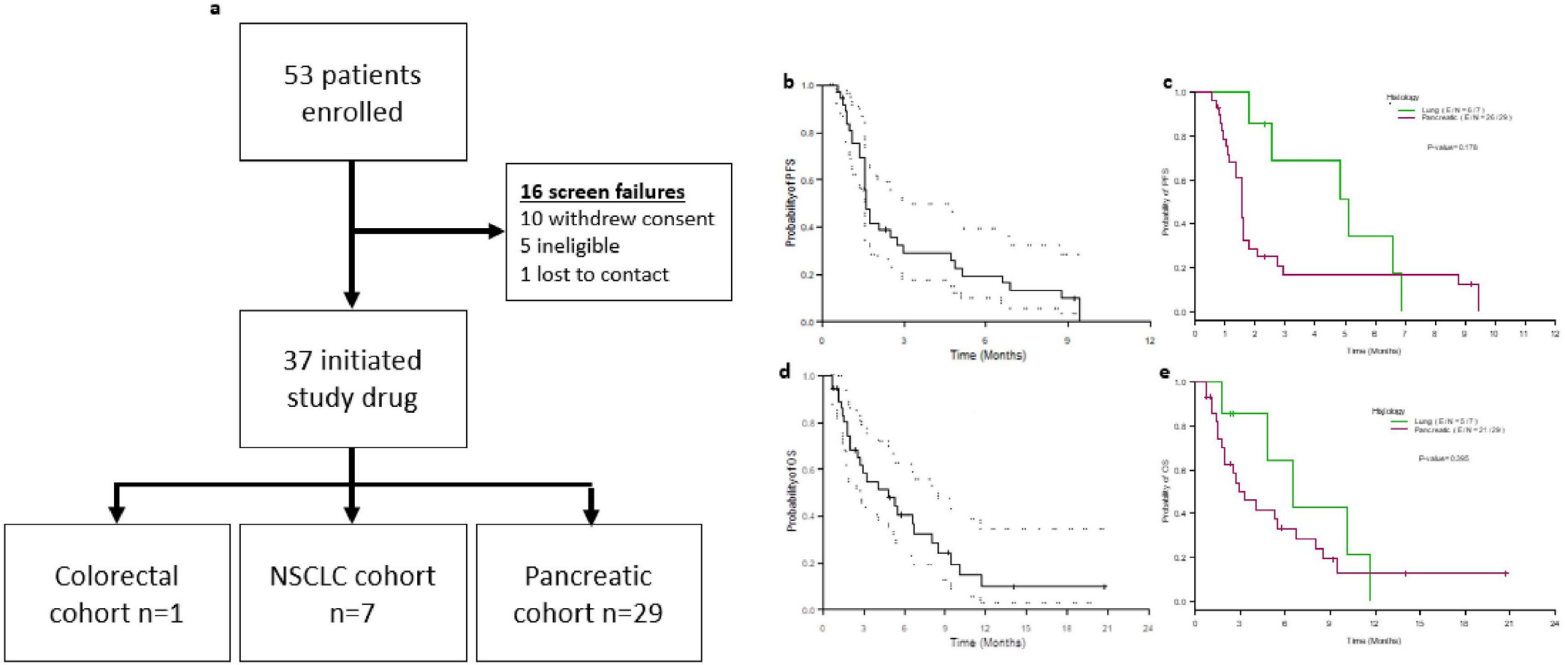
Clinical enrolment and outcomes from the phase II trial. (**A**) Clinical trial diagram showing patient enrolment and cohort allocation. (**B and C**) Kaplan-Meier curves showing progression-free survival among (**B**) all patients and (**C**) stratified by histology. (**D and E**) Kaplan-Meier curves showing overall survival among (**D**) all patients and (**E**) stratified by histology. NSCLC, non-small cell lung cancer.

Given the positive in vivo impact on survival in murine PDAC, we next investigated the clinical efficacy of this drug combination in a phase II clinical trial. 53 patients were enrolled between April 2017 and March 2020. There were 16 screen failures (figure 3A). Of the 37 patients who started treatment, 29 were in the pancreatic cancer cohort, 7 in the NSCLC cohort and 1 in the MRD CRC cohort. Demographic and relevant mutations are present in online supplemental table S3. The sex of patients on study was predominately male with a median age of 65 (IQR 59–72). The most common prior regiments used to treat PDAC patients were gemcitabine and abraxane (83%) followed by FOFIRNOX (83%), while NSCLC patients received carboplatin or cisplatin-based chemotherapy (71%) followed by PD-1 inhibition (57%) (online supplemental table S3).

In September 2020, the study was terminated as danvatirsen development was discontinued by the study sponsor due to the acquisition of this drug by another company. After a median follow-up of 14 months, 33 of 37 patients exhibited progressive disease, and 26 patients had died. No objective responses (partial or complete responses) were noted. The overall median progression-free survival (PFS) was 1.6 months (figure 3B), while PFS in the NSCLC and PDAC cohorts were 5.1 and 1.6 months, respectively (figure 3C). The median overall survival among all patients was 4.8 months (figure 3D), while overall survival in the NSCLC and pancreatic cancer cohorts was 6.6 and 3.3 months, respectively (figure 3E). Three of five deaths (60%) in the NSCLC cohort and 17 of 21 deaths (81%) in the PDAC cohort were post-progression.

The single MRD CRC was treated with FOLFOX +/− cetuximab, IROX with avastin, and pembrolizumab. This patient exhibited a best response of progressive disease and completed six cycles of systemic therapy.

Of seven patients enrolled in the NSCLC cohort, five patients (71%) exhibited a best response of SD. A median of six cycles (range 2–12) of systemic therapy were completed. As of last follow-up one patient was only observed with SD for 2.3 months and thus was not evaluable for the primary endpoint of 4-month DCR. Four of the six evaluable patients (66.7%, 95% CI 22.3% to 95.7%) met the primary endpoint. Even using a 95% CI, the lower limit was still higher than the null 4-month DCR (≤20%) and thus the null hypothesis was rejected at the two-sided significance level of 0.05, concluding that the treatment is promising.

29 patients were enrolled in the pancreatic cancer cohort. After enrolment of the first 12 patients, 4-month DC was observed in >1 patient, and thus the study proceeded to second-stage enrolment. The best response in the pancreatic cancer cohort was SD in 10 patients (34%). A median of three cycles (range 1–11) of systemic therapy were completed. As of last follow-up six patients had been observed with SD less than 4 months and were not evaluable for the primary endpoint of 4-month DCR. Among 23 evaluable patients 4 exhibited 4-month DC in the pancreatic cancer cohort (17.4%, 95% CI 5% to 38.8%). Even using a 95% CI, the lower limit was still higher than the null 4-month DCR (≤5%) and thus the null hypothesis was rejected at the two-sided significance level of 0.05, concluding that the treatment is promising.

Stratifying by sex did not reveal significant differences in PFS in both NSCLC (online supplemental figure S3A) and PDAC (online supplemental figure S3B) cohorts (both p >0.10). Among patients evaluable for the primary endpoint, within the NSCLC cohort one of two females (50%) and three of four males (75%; p=1.0) achieved 4-month DCR and within the PDAC cohort one of seven females (14%) and 3 of 16 males (19%; p=1.0) achieved 4-month DCR.

#### Adverse events

Treatment-related adverse events occurring in at least two patients are listed in online supplemental table S4. There were no grade 5 toxic effects. Two grade 4 toxic effects were observed, both of which were laboratory based (neutrophil and platelet count decrease), and 12 grade 3 toxic effects occurred. Treatment related discontinuation occurred in three patients (8%), which resulted in their withdrawal from the study due to toxicities after six, three and one cycles.

### Clinical trial translational correlatives

#### Liquid biopsy findings

Given the lower number of NSCLC and MRD CRC patients, translational analyses focused on the pancreatic cancer cohort. Of these 12 pancreatic patients tested, 4 exhibited baseline KRAS mutations detected through cfDNA and 2 patients exhibited KRAS mutations detected through exosome DNA. The presence of baseline KRAS mutation by either method was not associated with patient outcomes. Baseline mutation status assessed via tumour biopsies were obtained for 15 pancreatic cancer patients. Three patients were found to exhibit both TP53 mutations and SMAD4 mutations. The presence of TP53 or SMAD4 mutations (n=7) was associated with longer PFS (median 9.43 vs 1.31 months, log-rank p=0.034).

Among the entire cohort, 2 patients exhibited STK11 mutations, 1 with pancreatic cancer (290+1G>T) and another with NSCLC (721G>C). Both patients achieved a best response of SD. The pancreatic cancer and NSCLC patients with STK11 mutations were taken off study at 3 and 7 months, respectively, due to grade 3 transaminitis without evidence of progression of disease.

**Figure 4:**
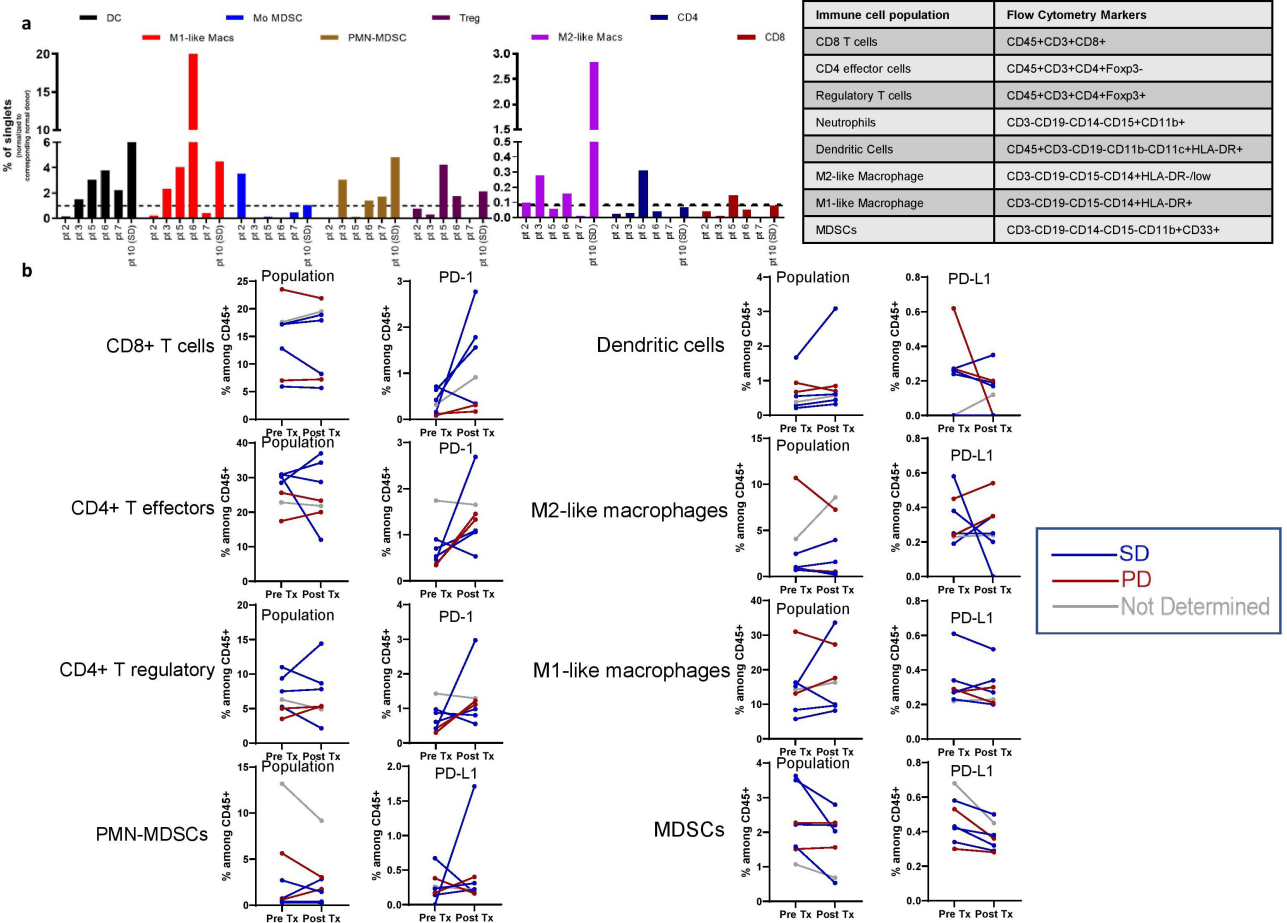
Flow cytometric analysis of tumour-infiltrating lymphocyte (TIL) and circulating peripheral blood cells. (**A**) Baseline TIL populations in six patients, one of whom achieved stable disease on trial. Right panel details the flow cytometry gating for each population. (**B**) Change in the percentage of (as a proportion from the CD45+ population) of circulating immune cell populations, those expressing STAT3+, and those expressing either PD-1 or PD-L1 between baseline and cycle 1 week 4 of systemic therapy. DC, dendritic cell; MDSCs, myeloid-derived suppressor cells; Mo, monocytic.

Flow cytometry was conducted on biopsy samples from six pancreatic cancer patients. Five experienced a best response of PD while one exhibited SD lasting 9 months. Interesting the one patient who achieved SD exhibited a higher proportion of dendritic cells than the other five patients (figure 4A). Flow cytometry noted trends in increased PD-1 expression on peripheral CD8 T cells and CD4 T effector and T regulatory cells (figure 4B).

### Postclinical trial laboratory studies

#### Pancreatic stellate cell activation and increased expression of immunosuppressive features following STAT3 ASO treatment

**Figure 5:**
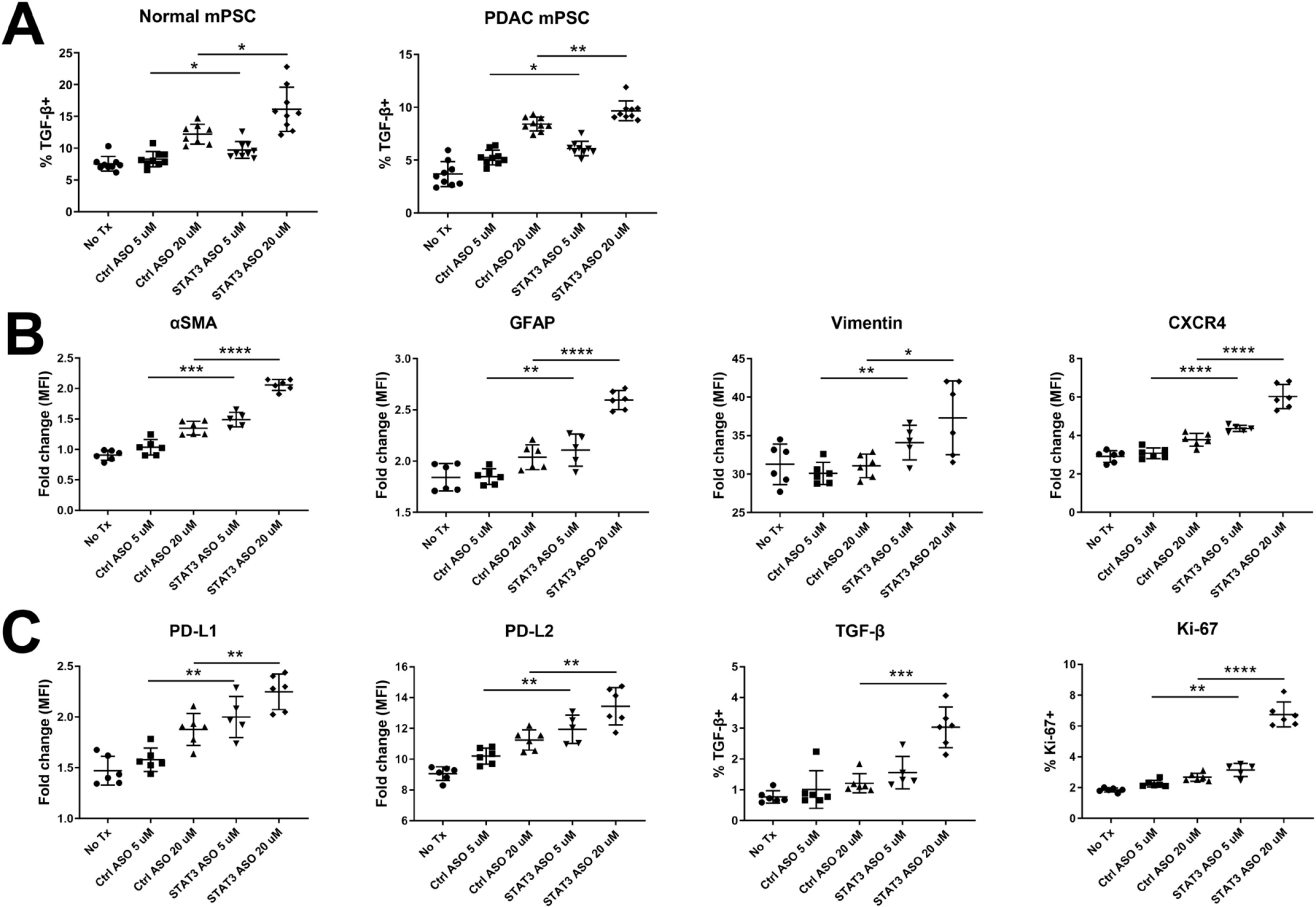
STAT3 ASO treatment fails to attenuate the immune-suppressive phenotype of PSCs in culture. Following treatment with STAT3 ASO for 4 days, normal and PDAC-associated murine PSCs (mPSCs) were stained for flow cytometric analysis of (**A**) TGF-β. Human PDAC-associated PSCs were treated similarly and stained for (**B**) PSC-associated activation markers and (**C**) expression of immune-suppressive molecules. Data shown are cumulative of three experiments. Statistical significance was calculated using one-way analysis of variance followed by Tukey multiple means post-test, with *p<0.05 and ****p<0.0001. MFI, mean fluorescence intensity. ASO, antisense oligonucleotide; PDAC, pancreatic adenocarcinoma; PSC, pancreatic stellate cell; STAT3, signal transducer and activator of transcription 3.

We treated murine and human pancreatic stellate cells (PSCs) with STAT3 ASO followed by flow cytometry phenotyping. Surprisingly, we found that normal and PDAC-associated murine PSCs increased their production of TGF-β as measured by LAP staining when treated with the STAT3 ASO (figure 5A). For PDAC-derived human PSCs, we found upregulated αSMA, GFAP, Vimentin and CXCR4 following treatment with 5 µM and 20 µM STAT3 ASO (figure 5B). In addition to the fibroblast activation indicated by these markers, CXCR4 expression by PSCs has been shown to facilitate invasiveness in PDAC.^18^ STAT3 ASO treatment also promoted, rather than reduced, expression of the immunosuppressive molecules PD-L1, PD-L2 and TGF-β (only at 20 µM) and increased the percentage of Ki67 expression (figure 5C). To ensure STAT3 ASO was working at a molecular level, we validated its capacity to knock down both total and phosphorylated STAT3. We found that the ASO reduced levels of both forms of STAT3 in a dose-dependent manner (online supplemental figure S4).

## Discussion

We present here a co-clinical trial and basic science experiment investigating combination therapy with STAT3 inhibition using a STAT3 ASO plus checkpoint inhibitors. Our in vivo experiments demonstrated that STAT3 ASO combined with checkpoint inhibitors improved survival in a mouse PDAC model compared with STAT3 ASO or checkpoint inhibitors alone. In parallel, a phase II clinical trial was conducted evaluating combination treatment with danvatirsen and durvalumab predominately in patients with PDAC and NSCLC. Median PFS in the NSCLC and PDAC cohorts were 5.1 and 1.6 months and overall survival in the NSCLC and PDAC cohorts were 6.6 and 3.3 months, respectively. Among the NSCLC cohort 66.7% (95% CI 22.3% to 95.7%) met the primary endpoint of 4-month DCR while in the PDAC cohort 17.4% (95% CI 5% to 38.8%) met the primary objective. As the 95% CIs for both cohorts exceeded the pre-specified null 4-month DCR threshold, this combination was deemed promising. Further mechanistic insight in the lack of response with this drug combination was provided by our in vitro evaluation of the myeloid compartment, which identified STAT3 ASO-induced activation of suppressive myeloid compartments, which may counteract simultaneous antitumour immune activation within the T cell compartment and may explain the lack of objective response on trial.

Danvatirsen has been tested in multiple early phase clinical trials. As a single agent, danvatirsen has shown evidence of objective response in lymphoma and a near partial response in NSCLC.^6^ Following this study, a phase Ib study in diffuse large B cell lymphoma identified a modest, 6.3%, rate of objective response.^19^ Combination danvatirsen and checkpoint inhibitors were tested for the treatment of solid tumours in the multi-arm phase Ib/2 SCORES study. One of these arms evaluated the activity of combination danvatirsen with durvalumab in PD-L1-naïve squamous cell head and neck cancer patients and identified a 25% rate of objective response.^11^ As most enrolled patients had PDAC, it is worth highlighting the central role of PSCs in this cancer type. The desmoplastic conditions created by PSCs may directly contribute to the lack of T cell infiltration into the tumour, which is a crucial step needed for the efficacy of ICB therapies. PSCs also secrete cytokines to recruit immature myeloid cells from the bone marrow, and patients with PDAC have increased numbers of circulating MDSCs in later stages of disease.^20 21^

To investigate the lack of benefit observed in the clinical trial, the effects of STAT3 ASO on myeloid-derived cells were analysed in vitro. We noted an increase in the immune suppression phenotype in these cell types following application of a STAT3 ASO. Our data concerning male vs female myeloid cell responses to STAT3 ASO treatment suggest that mice may have a dimorphic response based on sex. Our use of male mice for the survival studies and TIL analyses may only reflect the response of a subset of patients, and the immune response may differ in female mice. For example, female mice have been reported to have a more active immune response in terms of pro-inflammatory cytokine production, T cell activation, and type I interferon signalling.^22^ This appears to be in line with two recent studies that identified strong antitumour activity following STAT3 ASO treatment in CT26, a tumour model used in female mice.^12 23^ The clinical trial data did not identify such an association; however, there were only a minority of women patients enrolled on trial.

These preclinical findings showing that, in some cases, the STAT3 ASO can result in enhancement of suppressive myeloid and fibroblast function rather than producing the expected impairment based on STAT3 inhibitor and functional genetic attenuation studies should be taken into consideration in planning future clinical studies. Mechanistically, there can be reciprocal regulation of STAT3 and STAT1 and it is possible that the MDSC activation observed is the product of compensatory STAT1 activation in the setting of ASO ablation of STAT3. Future studies which further clarify this mechanism will aid in designing clinical studies with either STAT3 ASO dose regimens or therapeutic combinations designed to minimise the potential of this adverse stromal outcome.

Multiple limitations deserve mention. We did not complete enrolment of the planned patient number in the clinical trial. Although no objective response were noted, the combination has been deemed promising for future investigation based on the predetermined null hypotheses for the primary endpoint of 4-month DCR. Furthermore, we were unable to acquire a significant amount of tissues for correlative analysis on trial. Additional tissues would facilitate in-depth analyses of the myeloid compartment that could have shed valuable insight into mechanisms underlying the lack of objective response for this drug combination.

In summary, the combination of durvalumab and danvatirsen did not result in objective responses however it did meet our pre-specified threshold to show promise for these two cancer types. Based on our preclinical data, future iterations of trials employing STAT3 inhibition may achieve greater efficacy if rational combinations are developed or the drug is modified in a manner that reduces the immune suppressive effects via the myeloid compartment.

## Supplementary material

10.1136/bmjonc-2023-000133 online supplemental file 1

## Data Availability

Data are available upon reasonable request.
