# Supplementary material for: Preclinical study and parallel phase II trial evaluating antisense STAT3 oligonucleotide and checkpoint blockade for advanced pancreatic, non-small cell lung cancer and mismatch repair-deficient colorectal cancer
Source: BMJ Oncol. 2024 Jul 30;3(1):e000133. doi: 10.1136/bmjonc-2023-000133 (PMC11347683; doi:10.1136/bmjonc-2023-000133)
Supplement: online supplemental file 1 [file bmjonc-2023-000133supp001.pdf]

Supplementary Table 1. Flow cytometry antibody catalog

Target	Clone	Fluorophore	Source	Catalog No.	Use
CD45.2	30-F11	BUV395	BD Biosciences	564279	TIL
CD4	GK1.5	BUV496	BD Horizon	612952	TIL
CD8a	53-6.7	BV786	BD Biosciences	563332	TIL
CD3	17A2	BV711	BD Biosciences	740739	TIL
PD-1	29F.1A12	BV605	BD Biosciences	748267	TIL
CD11b	M1/70	A647	BioLegend	101251	TIL
CD11c	N418	Pe-Cy5	BioLegend	117316	TIL
Ly6G	1A8	BV421	BD Horizon	562737	TIL
Ly6C	HK1.4	APC-eF780	Thermo Fisher	47-5932-82	TIL
CD14	MϕP9	BUV395	BD Horizon	563561	Clin Trial TIL
CD8	RPA-T8	BUV496	BD Horizon	564805	Clin Trial TIL
CD3	UCHT1	BUV737	BD Horizon	564307	Clin Trial TIL
CD68	Y1/82A	BV421	BD Biosciences	564943	Clin Trial TIL
Ki-67	B56	BV510	BD Biosciences	563462	Clin Trial TIL
CD4	S3.5	BV605	Invitrogen	Q1008	Clin Trial TIL
CD11c	B-ly6	BV650	BD Horizon	563404	Clin Trial TIL
CD45RO	UCHL1	BV711	BD Horizon	563722	Clin Trial TIL
HLA-DR	G46-6	BB515	BD Biosciences	564516	Clin Trial TIL
CD33	P67.7	PerCP Cy5.5	BD Biosciences	341650	Clin Trial TIL
FoxP3	FJK-16s	AF532	eBioscience	58-5773-82	Clin Trial TIL
Arginase	658922	PE	R&D Systems	IC8026P	Clin Trial TIL
CTLA-4	BNl3	Pe-Cy5	BD Biosciences	555854	Clin Trial TIL
PD-L1	MlH1	Pe-Cy7	BD Biosciences	558017	Clin Trial TIL
CD15	Hl98	APC	BD Biosciences	551376	Clin Trial TIL
CD11b	M1/70	Alexa 700	Biolegend	101222	Clin Trial TIL
PD-1	J43	APC eFluor 780	Thermo Fisher	47-9985-82	Clin Trial TIL
CXCR4	2B11	efluor 450	Thermo Fisher	48-9991-82	mPSC/hPSC
Ki-67	B56	BV510	BD Biosciences	563462	mPSC/hPSC
PD-L1	MIH5	BV711	BD Biosciences	563369	mPSC
PD-L1	MIH1	BV650	BD Horizon	563740	hPSC
LAP	TW4-2F8	PerCP-Cy5.5	BD Pharmingen	562544	mPSC/hPSC
Arginase 1	8C9	Self-Conjugated	Santa Cruz	sc-47715	mPSC/MDSC
Arginase 1	polyclonal sheep	Alexa 700	R&D Systems	IC5868N	hPSC
Alpha-SMA	1A4	eFluor 570	eBioscience	41-9760-82	mPSC/hPSC
GFAP	GA5	eFluor 615	eBioscience	42-9892-82	mPSC/hPSC
PD-L2	TY25	APC	BD Pharmingen	560086	mPSC
PD-L2	MIH18	APC	BioLegend	345508	hPSC
Vimentin	polyclonal rabbit	APC-Alexa 750	Bioss Antibodies	bs-23063R-A750	mPSC/hPSC
CD86	GL1	BUV395	BD Biosciences	564199	MDSC
CD3	145-2C11	BUV496	BD Biosciences	564661	MDSC
CD80	16-10A1	BUV737	BD Horizon	564670	MDSC
Ly6G	1A8	BV421	BD Biosciences	562737	MDSC
Ki67	B56	BV510	BD Biosciences	563462	MDSC
CD206	C068C2	BV605	BioLegend	141721	MDSC

MHCII	M5/114.15.2	BV650	BD Horizon	563415	MDSC
PD-L1	MIH5	BV711	BD Biosciences	563369	MDSC
NOS2	CXNFT	R-PE	eBioscience	12-5920-82	MDSC
LAP	TW7-16B4	PE-Cy7	eBioscience	25-9821-82	MDSC
Cd68	Y1/82A	Alexa 647	BioLegend	333819	MDSC
CD11b	M1/70	Alexa 700	Thermo Fisher	56-0112-82	MDSC
Ly6C	HK1.4	APC-eFluor 780	eBioscience	47-5932-82	MDSC
Antibody labeling kit		Alexa Fluor 594	Thermo Fisher	A20185	mPSC
Antibody labeling kit		Alexa Fluor 700	Thermo Fisher	Z25011	MDSC

Supplemental Table 2: Number of animals for mT4 PDAC therapy study.

Experimental Group	Total Mice	n	Median OS (days)
Untreated	15	2	17
Control ASO	10	2	18.5
STAT3 ASO	10	2	20
Anti-CTLA-4/Anti-PD-1	16	2	27.5
Control ASO + Anti-CTLA-4/Anti-PD-1	5	1	21
STAT3 ASO + Anti-CTLA-4/Anti-PD-1	10	2	38

Supplementary Table 3. Trial Patient Characteristics

Variable (Cohort)	Category	Entire Cohort (n=37)	PDAC (n=29)	NSCLC (n=7)
Age, median (IQR)		65 (59-72)	69 (60-72)	69 (60-78)
Gender	Female	11 (30%)	9 (31%)	2 (21%)
	Male	26 (70%)	20 (69%)	5 (79%)
KRAS mutation	NA	16 (43%)		
	No	8 (22%)		
	Yes	13 (35%)		
TP53 mutation	NA	16 (43%)		
	No	12 (32%)		
	Yes	9 (24%)		
SMAD4 mutation	NA	16 (43%)		
	No	18 (49%)		
	Yes	3 (8%)		
MSS deficient	NA		23 (79%)	
	No		6 (21%)	
	Yes		0	
BRCA mutation	NA		11 (38%)	
	No		16 (55%)	
	Yes		2 (7%)	
EGFR mutation	NA			1 (14%)
	No			4 (57%)
	Yes			2 (29%)
Alk alteration	NA			2 (29%)
	No			5 (71%)
	Yes			0
PD-L1 positive	NA			1 (14%)
	No			3 (43%)
	Yes			3 (43%)
Prior Systemic Therapies	FOLFIRINOX		24 (83%)	
	Gemcitabine and Abraxane		25 (86%)	
	Capecitabine		8 (28%)	
	PD-1 inhibition			4 (57%)
	Carboplatin or Cisplatin-based Chemotherapy			5 (71%)
	Tyrosine Kinase Inhibitor			2 (29%)

Abbreviations: PDAC: pancreatic ductal adenocarcinoma, NSCLC: non-small cell lung cancer, MSS: microsatellite stable, FOLFIRNOX: folinic acid, fluorouracil (5-FU), irinotecan, and oxaliplatin. Note as systemic therapies listed including multiple different lines for each patient, summation of frequencies and percentages from these rows exceeds the total number of patients.

Supplementary Table 4. Treatment-related adverse events with 2 or more patients

Adverse event	Grade 1 No. (%)	Grade 2 No. (%)	Grade 3 No. (%)	Grade 4 No. (%)	Grade 5 No. (%)	All grades No. (%)
Platelet count decrease	11 (29.73)	4 (10.81)	3 (8.11)	1 (2.7)	0 (0)	19 (51.35)
ALT increase	1 (2.7)	3 (8.11)	2 (5.41)	0 (0)	0 (0)	6 (16.22)
AST increase	2 (5.41)	2 (5.41)	0 (0)	0 (0)	0 (0)	4 (10.81)
ALT and AST increase	7 (18.92)	4 (10.81)	5 (13.51)	0 (0)	0 (0)	16 (43.24)
Fatigue	4 (10.81)	3 (8.11)	0 (0%)	0 (0)	0 (0)	7 (18.92)
Anemia	0 (0)	3 (8.11)	1 (2.7)	0 (0)	0 (0)	4 (10.81)
Diarrhea	4 (10.81)	0 (0)	0 (0)	0 (0)	0 (0)	4 (10.81)
Neutrophil count decrease	1 (2.7)	1 (2.7)	1 (2.7)	1 (2.7)	0 (0)	4 (10.81)
White blood cell decrease	2 (5.41)	2 (5.41)	0 (0)	0 (0)	0 (0)	4 (10.81)
Nausea and vomiting	3 (8.11)	0 (0)	0 (0)	0 (0)	0 (0)	3 (8.11)
Blood bilirubin increase	3 (8.11)	0 (0)	0 (0)	0 (0)	0 (0)	3 (8.11)
Mucositis oral	2 (5.41)	0 (0)	0 (0)	0 (0)	0 (0)	2 (5.41)
Nausea	1 (2.7)	1 (2.7)	0 (0)	0 (0)	0 (0)	2 (5.41)
Creatinine increase	1 (2.7)	1 (2.7)	0 (0)	0 (0)	0 (0)	2 (5.41)
Rash acneiform	2 (5.41)	0 (0)	0 (0)	0 (0)	0 (0)	2 (5.41)

Abbreviations: ALT, alanine aminotransferase; AST, aspartate aminotransferase.

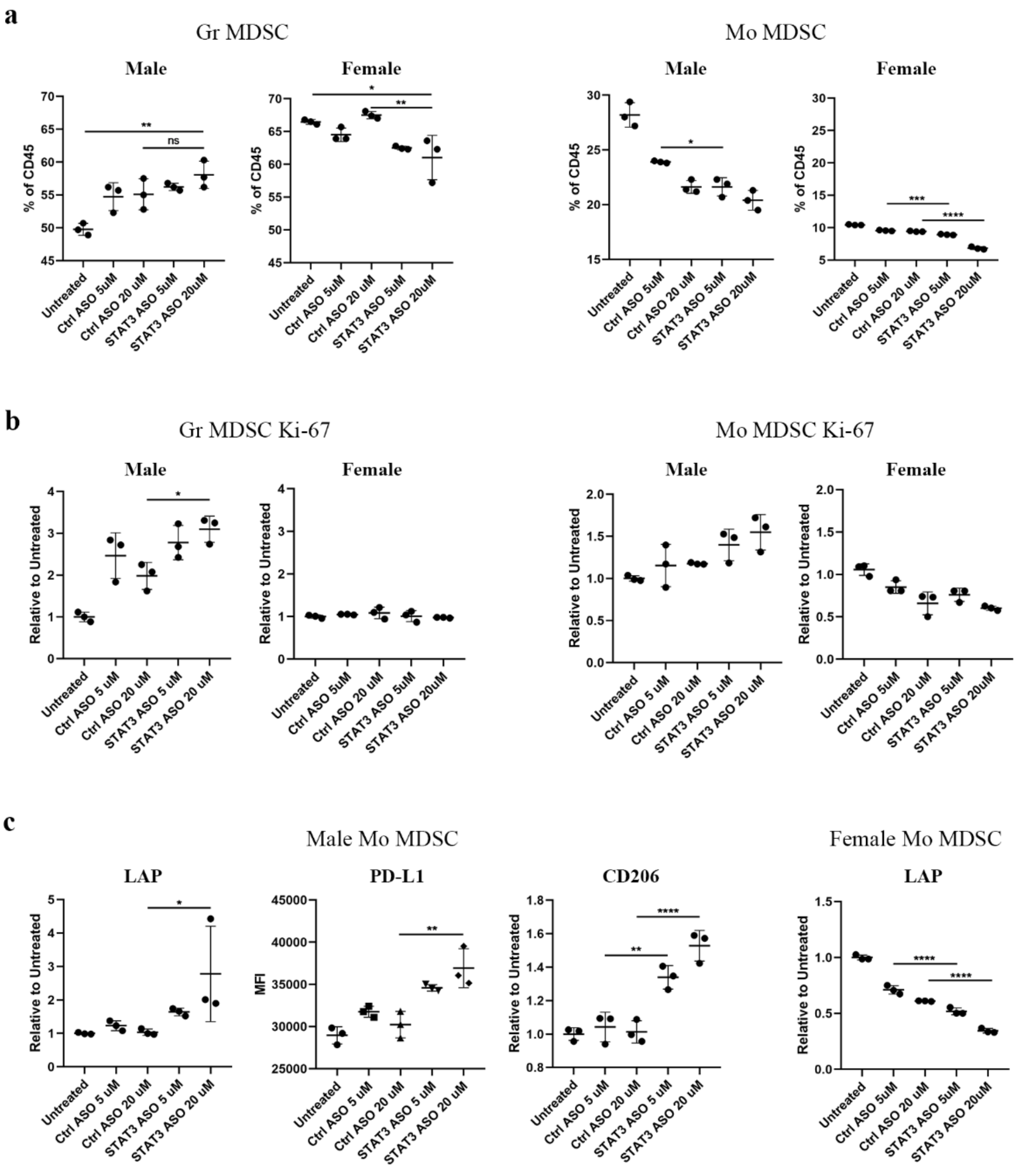

Supplementary Figure 1: Male and female MDSCs differ in their response to STAT3 ASO treatment**.** Bone marrow from wild-type male and female mice were cultured in GM-CSF and IL-6 as well as STAT3 or control ASO and stained for flow cytometric analysis. **(A)** percentages of granulocytic MDSCs (left) and monocytic MDSCs (right) were compared between male and female mice. **(B)** Proliferation of these cells was measured by Ki-67 staining. **(C)** Expression of immune suppressive molecules LAP, PD-L1, and CD206 are shown. Data shown are representative of 2 independent experiments. Statistical significance was calculated using 1-way ANOVA followed by Tukey multiple means post-test, with *$p<0.05$, **$p<0.005$, ***$p<0.0005$, and ****$p<0.0001$.

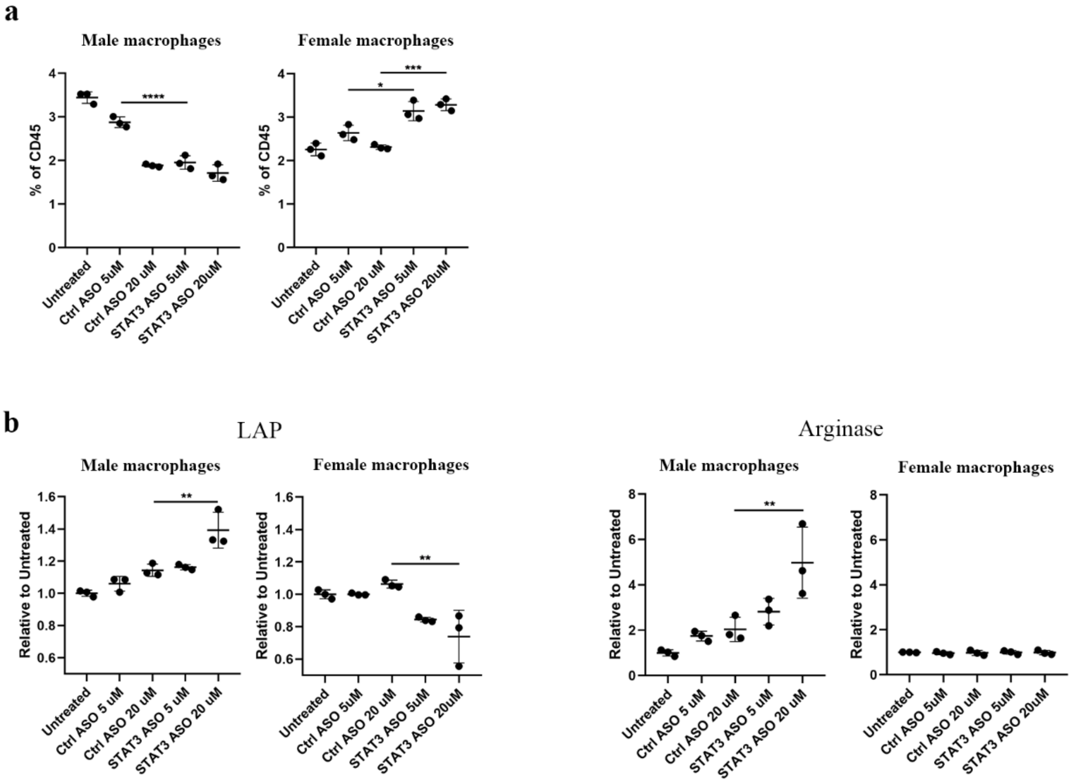

Supplementary Figure 2. Male and female macrophages differ in their response to STAT3 ASO treatment. Bone marrow from wild-type male and female mice were cultured in GM-CSF and IL-6 as well as STAT3 or control ASO and stained for flow cytometric analysis. Percentages of macrophages were compared **(A)** and expression of immune suppressive molecules LAP (left) and arginase (right) are shown in **(B)**. Data shown are representative of 2 independent experiments. Statistical significance for were calculated using 1-way ANOVA followed by Tukey multiple means post-test, with *$p<0.05$, **$p<0.005$, ***$p<0.0005$, and ****$p<0.0001$.

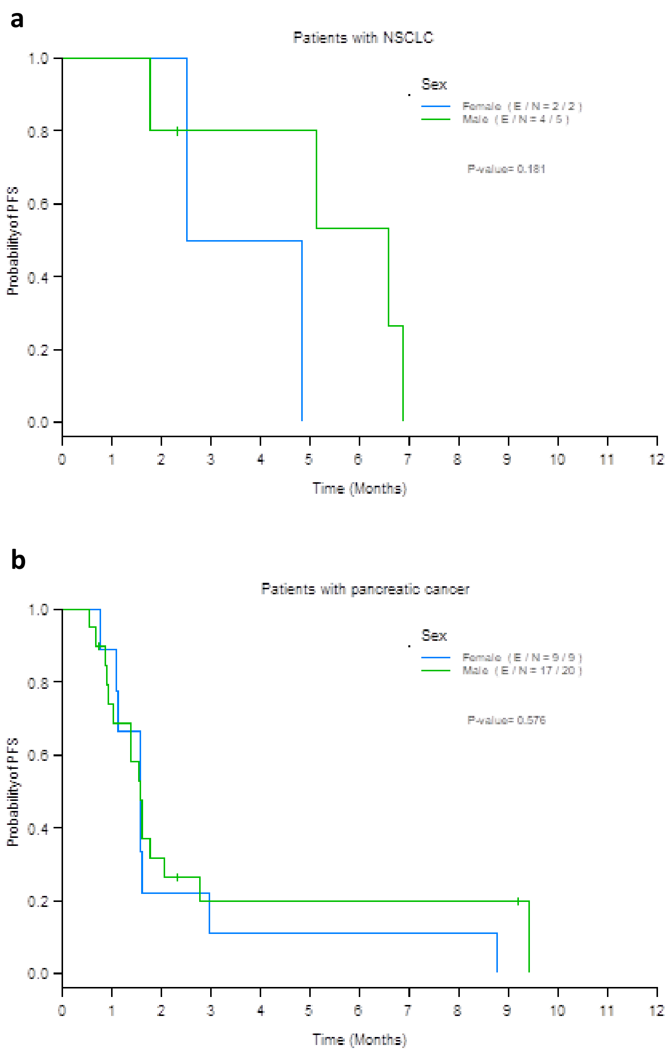

Supplementary Figure 3. Progression free survival (PFS) stratified by sex for patients with non-small cell lung cancer (NSCLC) (**A**) and pancreatic cancer (**B**).

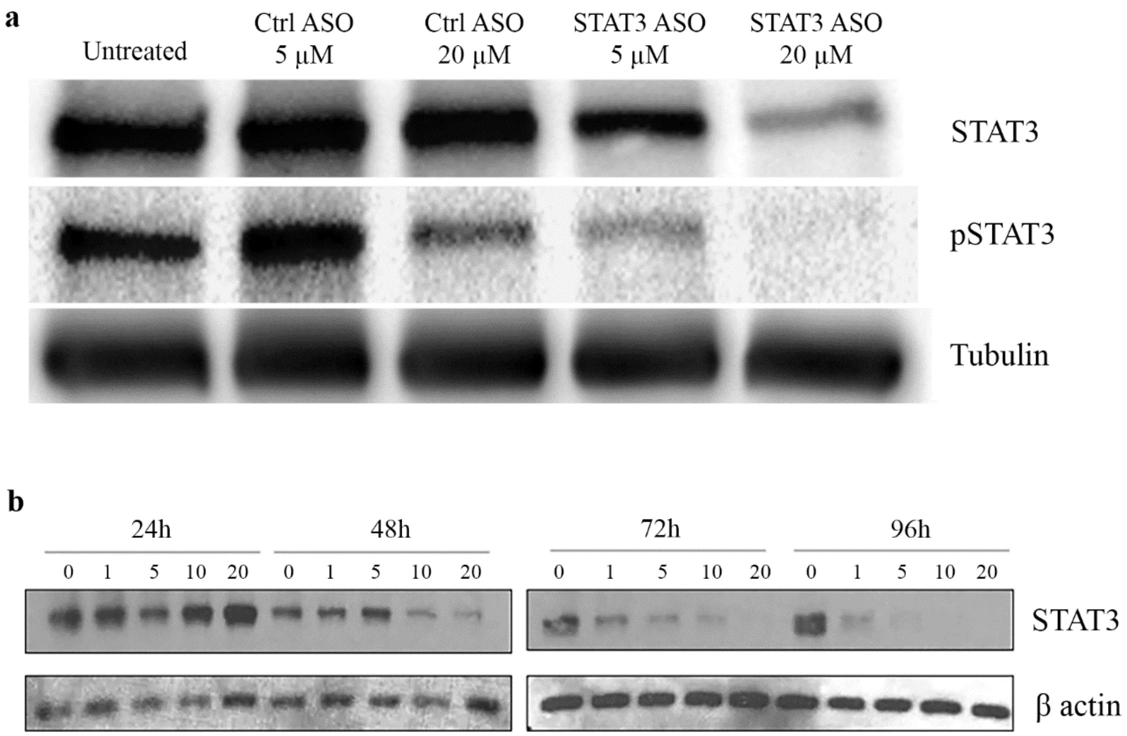

Supplementary Figure 4. STAT3 levels after STAT3 ASO treatment. Control (Ctrl) and STAT3 ASO treatment at 5 and 20 μM for 4 days reduced STAT3 and phosphorylated STAT3 (pSTAT3) protein levels in a **(A)** human PSC cell line and **(B)** STAT3 levels in primary mouse MDSCs assayed using via western blot. Reduction in pSTAT3 levels observed at 20 μ M Ctrl ASO attributed to either off target activation of immune sensors by Ctrl ASO or reduction in PSC viability.
